# UFMylation System: Biological Functions, Molecular Mechanisms, Diseases, and Drug Discovery

**DOI:** 10.1002/mco2.70424

**Published:** 2025-10-09

**Authors:** Huiyan Li, Fei Meng, Junjie Liang, Yijie Wang, Changliang Shan, Yan Chen

**Affiliations:** ^1^ Institute of Biomedical Sciences Shandong Provincial Key Laboratory of Animal Resistance Biology Collaborative Innovation Center of Cell Biology in Universities of Shandong Center For Cell Structure and Function College of Life Sciences Shandong Normal University Jinan China; ^2^ Department of Hepatobiliary Surgery The First Affiliated Hospital of Jinan University Guangzhou China; ^3^ College of Pharmacy Nankai University Tianjin China

**Keywords:** diseases, drugs, post‐translational modification, signaling pathways, UFMylation

## Abstract

UFMylation, a novel ubiquitin‐like modification, plays a critical role in various intertwined cellular processes, such as the immune response, DNA damage repair, unfolded protein response (UPR), autophagy, endoplasmic reticulum (ER)‐phagy, stem cell self‐renewal, apoptosis, and metastasis. Dysfunction of UFMylation has been implicated in a variety of human diseases, including neurogenesis, hematopoiesis, liver development, and cancer. While this field is just emerging, research on UFMylation has escalated rapidly in recent years, with great advances having been made. However, only a few substrates of UFMylation have been identified so far, and the biological functions as well as the molecular mechanisms of the UFMylation system in tumorigenesis and the tumor microenvironment remain poorly understood. In this review, we first summarize current knowledge of the components, biochemical peculiarities, and working principles of the UFMylation system. Second, we provide a multidisciplinary review of the cellular functions, molecular mechanisms, and pathophysiological roles of the UFMylation system, with a particular emphasis on the intricate relationship between UFMylation and cancer. Finally, we discuss the potential of targeting UFMylation in cancer treatment and highlight outstanding questions for future investigation in this field.

## Graphical abstract

UFMylation, a novel ubiquitin‐like modification, plays an essential role in various cellular processes, such as the immune response, DNA damage repair, unfolded protein response (UPR), autophagy, endoplasmic reticulum (ER)‐phagy, stem cell self‐renewal, apoptosis, and metastasis. Dysfunction of UFMylation has been implicated in a variety of human diseases, including cancer, making it an attractive target for cancer therapy.

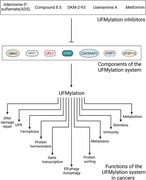

## 1 Introduction

Post‐translational modifications (PTMs) play pivotal roles in protein homeostasis, allowing proteins to acquire crucial functions [1]. In addition to phosphorylation, research on ubiquitin (Ub) and Ub‐like modifications is escalating rapidly [2, 3]. A variety of Ub‐like modified proteins (UBLs), such as SUMO [4, 5], NEDD8 [6, 7], ISG15 [8], Atg8 [9, 10, 11], Atg12 [12], Urm1 [13, 14], Fat10 [15], Hub1 [16], UFM1 [17, 18, 19, 20], and FUB1 [21], are conjugated to target proteins or lipids through the catalytic action of E1 activating enzymes (E1s), E2 conjugating enzymes (E2s), and E3 ligases (E3s). Ultimately, these modifications regulate the stability, biological activity, and function of the target molecules [22, 23]. Among these UBLs, the discovery of Ub‐fold modifier 1 (UFM1) has stimulated intense research into the molecular mechanism and the biological functions of UFMylation [17, 18, 19, 20].

**FIGURE 1:**
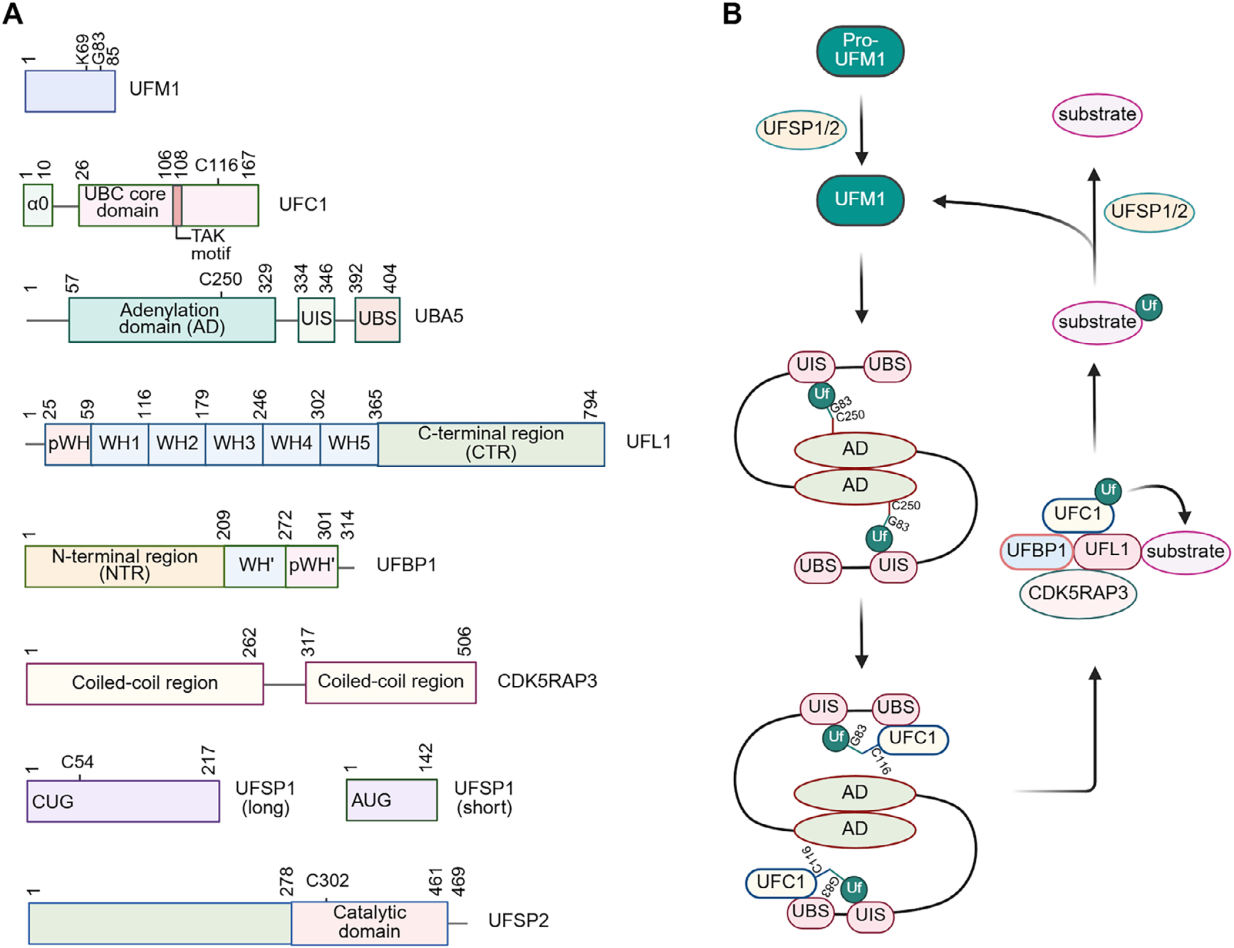
**Overview of the UFMylation system**. (**A**) Key residues and structural domains of the core components of the UFMylation system, including UFM1 (also referred to as Uf), UBA5, UFC1, UFL1, UFBP1 (also known as DDRGK1), CDK5RAP3, and UFSP1/2. UFSP1 has a long and a short isoform, translation of which starts from the CUG codon and the AUG codon, respectively. UIS, UFM1‐interacting sequence; UBS, UFC1‐binding site; WH, winged helix; pWH, partial winged helix. (**B**) The processes of UFMylation and deUFMylation. Pro‐UFM1 is catalytically cleaved by UFSP1/2 to expose the glycine 83 (G83) residue, generating mature UFM1. UBA5 forms a homodimer to stimulate the simultaneous association of UFM1 with the UIS of one UBA5 subunit and the cysteine 250 active site (C250) within the AD of the other subunit, leading to UFM1 activation. Next, UFM1 is transferred to UFC1 bound to the UBS of the other UBA5. Finally, UFL1, together with UFBP1 and CDK5RAP3, promotes the conjugation of UFM1 to the substrate. UFMylation is a highly dynamic process, and UFM1 can be removed from substrates by UFSP1 and UFSP2 for recycling.

Two decades of research have uncovered the core components of the UFMylation system and its processes (Figure 1A,B). The first three key members of the UFMylation system, comprising UFM1, UBA5 (Ub‐like modifier activating enzyme 5), and UFC1 (Ub‐fold modifier‐conjugating enzyme 1), were discovered in 2004 by yeast two‐hybrid screening and LC‐MS/MS analysis [24, 25]. Biochemically, UBA5 and UFC1 function as UFM1‐activating and ‐conjugating enzymes, respectively [24, 26]. The single E3 ligase of the UFMylation system is UFL1 (UFM1‐specific ligase 1), which was isolated through LC‐MS/MS analysis aimed at identifying UFM1‐interacting proteins in 2010 [27, 28]. However, UFL1 on its own is unstable and inactive [29]. UFBP1 (UFM1 binding protein 1) and CDK5RAP3 (CDK5 regulatory subunit‐associated protein 3) are essential for UFL1 stability, activity, and substrate specificity [17, 18, 20]. UFMylation is a highly dynamic process [30]. UFSP1/2 (UFM1‐specific protease 1/2) removes UFM1 from UFMylated substrates to prevent excessive UFMylation [31]. Moreover, UFSP1/2 contributes to UFM1 maturation [32, 33, 34]. These core components of the UFMylation system possess conserved structures and functions in most eukaryotes, but not in fungi [20, 26], suggesting crucial roles for the UFMylation system in multicellular organisms. Recent studies have further implicated the UFMylation system in various biological processes, such as DNA damage repair, endoplasmic reticulum (ER) stress response, autophagy, lipid metabolism, gene expression, ribosomal assembly, stem cell pluripotency, immunity, and apoptosis [17, 18, 20]. Thus, dysfunction of the UFMylation system can result in multiple human diseases, including cancer.

This review systematically summarizes the biochemical characteristics of UFMylation, its multifaceted roles in disease progression, and the molecular mechanisms underlying its involvement in key pathways, such as estrogen receptor signaling, ferroptosis suppression, ribosomal function, and genome stability. We highlight the dual roles of UFMylation in tumor immunity, including its regulation of PD‐1/PD‐L1 axis and antiviral responses, and discuss its impact on cancer cell stemness and metastasis. Furthermore, we explore the therapeutic potential of targeting UFMylation components, emphasizing recent developments in small‐molecule inhibitors and diagnostic biomarkers. Outstanding questions, such as the crosstalk between UFMylation and other post‐translational modifications, the identification of UFMylation “readers”, and the context‐specific effects of UFMylation in different diseases, are critically evaluated. This comprehensive overview aims to bridge existing knowledge gaps and inspire future research directions in UFMylation‐related disease biology and therapy.

## 2 Components and Processes of the UFMylation System

Similar to other UBL conjugation enzymatic reactions, UFMylation is catalyzed by three enzymes: E1, E2, and E3. Here, we provide an overview of the UFMylation process (Figure 1A,B) [20]. The initial step involves the catalytic removal of the C‐terminal Ser‐Cys of UFM1 by UFSP1 and UFSP2, exposing its glycine residue to achieve maturation [35, 36]. Mature UFM1 is conjugated to and activated by UBA5. Subsequently, the ligase UFC1 accepts the activated UFM1 via trans‐thioesterification, facilitating the conversion of the thioester‐bound UFM1 and its ligation with UFC1 [37, 38, 39]. Finally, the substrate and the UFC1‐UFM1 intermediate are recruited to adjacent positions by UFL1 and UFM1 is transferred to the substrate [29]. UFL1 cannot act independently; it forms a complex with UFBP1 and CDK5RAP3, which are critical for UFL1 stability and activity [40]. UFMylation is a highly dynamic process, and UFM1 can be removed by UFSP1 and UFSP2 [19, 41].

### 2.1 UFM1 Maturation

UFM1, the central constituent of the UFMylation system, shares numerous structural characteristics with Ub in the ubiquitination pathway. UFM1 is conserved across various species, except in yeast [42]. It comprises 85 amino acids, and its mature form is achieved through cleavage of the last two amino acids (Ser‐Cys) (Figure 1A) [24]. The deUFMylation enzymes UFSP1 and UFSP2 play pivotal catalytic roles in this process, generating a mature C‐terminal Gly (Figure 1A,B), which forms isopeptide bonds with lysine residues on target proteins [32, 33, 34]. Unlike Ub and other UBLs, UFM1 features Val and Gly as its two terminal amino acids, rather than Gly‐Gly [23]. In addition, unlike Ub, which can generate different types of homotypic chains and branched chains to form poly‐ubiquitination by linking its own lysine residues, UFM1 primarily forms mono‐UFMylation [43]. Despite K69‐linked UFM1 chains being reported (Figure 1A), the remaining five lysine residues (K3, K7, K19, K34, and K41) do not participate in chain formation in cells [44]. Notably, K7 and K69 linkages have been observed in cell‐free reconstitution assays [29]. Although the sequence identity between UFM1 and Ub is only 15%, UFM1 possesses a Ub‐like fold comprising four β‐strands and two α‐helices and binds substrates similarly to Ub [24, 45, 46, 47]. It is worth noting that UFM1 mainly localizes in the nucleus [24], suggesting that UFMylation may predominantly occur on nuclear proteins and consequently regulates nuclear protein‐mediated biological processes, such as DNA damage repair, gene expression, and chromatin assembly.

### 2.2 UBA5 and UFM1 Activation

After maturation, UFM1 is activated by UBA5, which possesses a C‐terminal tail and an adenylation domain (AD, aa57–329) (Figure 1A). The AD comprises the ATP‐binding pocket and the catalytic cysteine 250 active site (Cys250), whereas the C‐terminal tail consists of a linker region flanked by a UFM1‐interacting sequence (UIS, aa334–346) and a UFC1‐binding site (UBS, aa392–404) (Figure 1A) [38, 48, 49]. Therefore, UBA5 is considered a non‐canonical E1 enzyme because its Cys250 is embedded within the AD. UBA5 consists of 404 amino acids, making it significantly smaller than other E1 enzymes [38]. Consequently, the catalytic mechanism of UBA5 differs from that of other E1 enzymes. UFM1 activation by UBA5 requires only two steps, while other E1 enzymes follow a three‐step mechanism. In the first step, the AD of UBA5 binds ATP, releasing a pyrophosphate and resulting in an adenylated UFM1 (UFM1‐AMP) intermediate [24]. In the next step, Cys250 of UBA5 launches a nucleophilic attack on the C‐terminal glycine of UFM1, releasing AMP, allowing UBA5 to bind to UFM1 through a thioester bond, forming a binary complex (UBA5–UFM1) to complete UFM1 activation (Figure 1B) [24, 50]. Notably, UBA5 homodimerization is indispensable for UFM1 activation, while UFM1 simultaneously associates with the UIS of one UBA5 subunit and the AD of the other subunit (Figure 1B) [48, 49]. This trans‐binding mechanism stabilizes the UBA5 homodimer and enhances ATP binding to UBA5 but is not observed in any other E1 enzyme [48, 51]. Nevertheless, the trans‐binding mechanism provides an attractive therapeutic target to modulate UBA5 activity.

While the adenylation activity and Cys250 are crucial for UBA5's catalytic function, the last 20 residues outside the AD are essential for its binding to UFC1 and subsequent UFM1 transfer [52, 53]. In humans, the long isoform of UBA5 not only contains an AD but also has an N‐terminal extension (NTE, aa1–56), which is not required for UFMylation but enhances the interaction of the AD with ATP. This results in an ATP to UBA5 ratio of 1:1 rather than 1:2, thereby increasing UFM1 activation at low ATP concentration [54]. Intriguingly, the arginine 55 to histidine (R55H) mutation, which decreases UBA5 activity, has been reported for individuals with severe infantile‐onset encephalopathy [55]. Although UBA5 deficiency leads to severe physiological defects, such as impaired megakaryocyte and erythrocyte differentiation in mice, resulting in severe anemia [56], overexpression of UBA5 also produces adverse effects similar to UBA5 deficiency. In such cases, UFM1 is transferred back from UFC1 to UBA5 due to the reversal of the trans‐thiolation reaction, impairing the cell's migratory ability [57]. However, how cells maintain optimal UBA5 activity remains to be investigated.

UBA5 predominantly localizes in the cytoplasm and is recruited to the ER by GABARAP proteins [58, 59]. Notably, UBA5 also displays nuclear distribution, especially when it is co‐transfected with another UBL, SUMO2, indicating that UBA5 may exert specific functions in the nucleus [59]. The function and underlying mechanism of the nuclear UBA5 need to be deciphered in future studies.

### 2.3 UFC1 and UFM1 Conjugation

UFC1, the unique E2 enzyme identified in the UFMylation system, is a protein composed of 167 amino acid residues with a molecular weight of 19.4 kDa [24]. UFC1 possesses low sequence homology with other E2 enzymes and lacks the His‐Pro‐Asn (HPN) catalytic motif that is conserved in other E2 enzymes [60, 61]. Instead, the HPN motif is replaced by a Thr‐Ala‐Lys (TAK, aa106–108) motif upstream of the catalytic cysteine 116 (Cys116) in UFC1 (Figure 1A) [29, 60]. T106I and K108A mutations dramatically impair UFMylation [29]. Additionally, UFC1 lacks the C‐terminal alpha‐helix and a conserved negatively charged residue that activates the substrate lysine during conjugation, unlike other E2 enzymes [29, 62]. These findings reveal that UFC1 potentially utilizes a unique mechanism for UFM1 conjugation, distinguishing it from other E2 enzymes.

The UBS of UBA5 binds to the hydrophobic pocket of UFC1 located at the opposite surface of the catalytic Cys116 to bring UFM1 bound to the AD of one UBA5 and UFC1 bound to the UBS of the other UBA5 into close proximity, thereby facilitating the trans‐thioesterification reaction and the transfer of UFM1 (Figure 1B) [52]. Hence, UFM1's transfer from UBA5 to UFC1 depends on UBA5 homodimerization. Notably, the hydrophobic pocket of UFC1 also interacts with the N‐terminal UBS of UFL1 [63, 64, 65], suggesting that UFL1 displays a higher affinity for UFM1 compared to discharged UBA5, ensuring efficient UFM1 transfer from UBA5 to UFL1. Interestingly, both the NTE of UBA5 and the linker region between the UIS and the UBS of UBA5 are critical for UFM1 transfer from UBA5 to UFC1 [52, 54]. The mechanisms of NTE‐ and linker region‐mediated UFM1 transfer remain elusive.

In addition to its canonical Ub‐conjugating (UBC) core domain, UFC1 has a conserved α‐helix (α0) at its N‐terminus, which is not present in other E2 enzymes. α0 adopts various conformations to accommodate different substrates (Figure 1A) [26, 66]. Importantly, UFC1 lacking α0 exhibits stronger UFMylation activity compared to the wild‐type (WT) UFC1 in vitro [34], indicating an inhibitory effect of α0 on UFM1 conjugation. K122, situated near Cys116 in UFC1, has been shown to be constitutively UFMylated, reducing the efficiency of UFM1 transfer [34]. Further work is required to elucidate the mechanism of α0's inhibitory effect on UFC1.

### 2.4 UFL1 E3 Ligase Complex and UFM1 Ligation

UFL1, also referred to as Maxer and RCAD, is currently the sole identified E3 enzyme in the UFMylation system, responsible for catalyzing the transfer of UFM1 to the lysine residues of substrates. It comprises 794 amino acids, with a molecular mass of approximately 90 kDa (Figure 1A) [27]. UFL1 is predominantly localized on the cytoplasmic surface of the ER membrane via its transmembrane domain [67]. Additionally, it contains a nuclear localization signal (NLS) that facilitates its transport to the nucleus [49, 67]. UFL1 lacks the conserved domains of other E3 enzymes, such as the really interesting new gene (RING) domain, E6AP carboxyl terminus (HECT) domain, or ring between ring fingers (RBR) domain [3], implying that UFL1 is a non‐canonical E3 ligase. Moreover, none of the single cysteine to alanine mutations affect the auto‐UFMylation of UFL1 or UFMylation of substrates [29], suggesting that no catalytic cysteine exists in UFL1. These findings indicate that UFL1 is a scaffold E3 ligase, similar to RING E3 ligases, which cooperate with E2 ligases to transfer Ub/UBL from E2 ligases to a lysine residue within substrates [3, 18].

So far, UFL1 is considered the unique E3 ligase in the UFMylation system. However, UFL1 on its own is unstable and inactive [29], indicating that UFL1 requires the assistance of other proteins for UFMylation. Indeed, several studies have shown that UFBP1 (also known as C20orf116, Dashurin, or DDRGK1) and CDK5RAP3 (also known as C53 or LZAP) are critical for UFL1 stability, activity, or substrate specificity (Figure 1A,B) [17, 18, 20]. Thus, these two proteins together with UFL1 are considered as core components of the E3 ligase complex in the UFMylation system. UFL1 possesses an N‐terminal helix (aa 1–25) followed by a partial winged helix (pWH) domain and five winged helix (WH) domains that extend into its C‐terminal region (CTR) comprising a stack of α‐helices (Figure 1A) [29]. Likewise, UFBP1 is composed of one N‐terminal transmembrane segment, a proteasome‐COP9‐initiation factor 3 (PCI) domain, one WH (referred to as WHʹ) domain, and one pWH (referred to as pWHʹ) domain (Figure 1A) [29, 63]. The pWH at the N‐terminus of UFL1 complements the pWHʹ at the C‐terminus of UFBP1 to form a composite WH (pWH‐pWHʹ) domain, thereby greatly enhancing UFL1 stability and activity [29, 63]. The N‐terminal helix (aa 1–25) of UFL1 functions as a UBS and associates with UFC1 similarly to UBA5 [65]. The UFL1‐UFBP1 complex bridges UFC1 and substrates on the cytosolic side of ER to induce aminolysis of the UFM1‐UFC1 thioester and subsequently promotes the conjugation of UFM1 to substrates [17, 18, 29, 63]. Although there is complete overlap between UFL1 and UBA5 in terms of their binding sites on UFL1, the affinity of UFL1 to UFC1 is sixfold higher than that of UBA5, ensuring efficient transfer of UFM1 from UBA5 to substrate [63]. Hence, the UFL1‐UFBP1 complex accounts for most UFMylation reactions [29, 63]. Notably, UFL1 possesses an NLS, and the WH domains recognize a spectrum of nucleic acids, indicating that the UFL1‐UFBP1 complex may engage in processes in the central dogma and epigenetic regulations [68].

CDK5RAP3, also known as C53 or LZAP, serves as a secondary adaptor protein for UFL1 (Figure 1A,B). In contrast to UFBP1 and UFL1, CDK5RAP3 is not essential for UFMylation; rather, it regulates the activity of the UFL1‐UFBP1 complex and determines substrate specificity [17, 29, 63]. Interaction with the UFL1‐UFC1 complex enhances CDK5RAP3 protein stability and its relocation to the ER, where CDK5RAP3 inhibits UFL1‐mediated UFMylation of UFBP1 at K267, thereby repressing UFMylation of H4, ASC1, and MRE11 [29, 63]. However, CDK5RAP3 promotes mono‐ and di‐UFMylation of ribosomal protein L26 (RPL26) by the UFL1‐UFC1 complex [58, 63]. The mechanism of CDK5RAP3's impact on substrate specificity needs further investigation in the future.

### 2.5 UFSP1/UFSP2 and deUFMylation

UFSPs, including UFSP1 and UFSP2, are novel proteases within the UFMylation system, characterized by cysteine‐based catalytic activity (Figure 1A,B). These proteases primarily convert the precursor form of UFM1 into its mature form by cleaving the C‐terminus of UFM1, removing two amino acid residues and exposing the conserved glycine. Additionally, UFSPs can remove UFM1 from UFMylated substrates, potentially preventing excessive UFMylation [31]. UFSPs are widely distributed: UFSP2 is present in most multicellular organisms including mammals, plants, nematodes, and Drosophila, while UFSP1 is absent in plants and nematodes. Initial studies suggested that UFSP1 is inactive in humans, indicating that UFSP2 processed pre‐UFM1 to mature UFM1. However, recent studies have shown that UFM1 can also be processed in cells lacking UFSP2, suggesting that other active proteases exist in the human UFMylation system. Using site‐directed mutagenesis, CRISPR/Cas9‐mediated genome editing, and mass spectrometry, researchers identified a longer form of UFSP1 translated from a non‐canonical CUG start site, in addition to the previously known short form (Figure 1A). This active UFSP1 not only matures UFM1 and initiates UFMylation but also removes the UFM1 modification from UFC1, potentially relieving its autoinhibition. Conversely, UFSP2 primarily removes UFM1 from RPL26. The distinct cellular localizations of UFSP1 (cytoplasm) and UFSP2 (ER membrane) may determine their functional differences [31, 34, 69]. UFMylation modification is a dynamic and reversible process. Notably, the compartmentalization of UFMylation components suggests spatial regulation of its functions, a feature that will be explored in the following sections through its disease‐relevant substrates.

## 3 Multifarious Functions of the UFMylation System in Cancer

The dual roles of UFMylation in cancer exemplify its context‐dependent functionality. Accumulating evidence positions UFMylation as a molecular switch that either promotes tumor progression or inhibits malignancy, with the outcome dictated by substrate specificity and cellular microenvironment (Table 1).

**TABLE 1:** UFMylation substrates involved in the cellular and molecular processes in cancer cells.

Substrates	Sites	Cell types	Functions	References
ASC1	K324/325/334/367	ER^+^ breast cancer cells: MCF‐7 and BT474.	Promotes p300‐SRC1‐ASC1 complex‐mediated ERα transactivation and ER^+^ breast tumor growth.	[149]
ERα	K171/180	ER^+^ breast cancer cells: MCF‐7 and BT474.	Prevents ERα from ubiquitination and degradation.	[150]
SLC7A11	ND	ER^+^ breast cancer cells: MCF‐7 and T47D.	Increases SLC7A11 stability and expression to suppress ferroptosis.	[162, 163]
PIR	K5/6	Pancreatic ductal adenocarcinoma cells: Tu8988t and PANC‐1.	Inhibits ferroptosis by increasing GPX4 transcription and decreasing the cytoplasmic transportation of HMGB1.	[164]
PLAC8	K103	TNBC cells: MDA‐MB‐231 and HCC1937.	Increases PLAC8 stability to attenuate PD‐L1 ubiquitination and degradation.	[133]
PD‐L1	K75/89/105/162/280/281	TNBC cells: MDA‐MB‐231. Melanoma cells: WM989 and B16F10. Mouse colon cancer cells: MC38. Liver cancer cells: Hepa1‐6. Bone osteosarcoma epithelial cells: U2OS.	Facilitates the ubiquitination and degradation of PD‐L1.	[135]
PD‐1	K210/233	T cells.	Inhibits the ubiquitination and proteasomal degradation of PD‐1, thereby enhancing immune suppression.	[136]
MAVS	K461	Gastric cancer cells: AGSiZ. Lymphoma cells: P3HR‐1.	Promotes lysosomal degradation of MAVS.	[137, 138]
RPL10	K30/101	Pancreatic adenocarcinoma (PAAD) cells: PANC‐1 and Mia PaCa‐2.	Promotes the expression of transcription factor KLF4 to increase cell proliferation and stemness.	[166]
PDK1	ND	Human gastric cancer cells: N87, AGS, MGC‐803, and HGC‐27.	Enhances PDK1 ubiquitination and degradation and alleciates PDK1‐AKT1‐GSK‐3β axis‐mediated EMT process and metastasis.	[168]
UFBP1	K267	ER^+^ breast cancer cells: MCF‐7. Liver cancer cells: HepG2.	Decreases IREα protein degradation and inhibits PERK activation, thereby inhibiting cell death.	[99 ]
		Human cervical carcinoma cells: HeLa. Colorectal adenocarcinoma cells: HT29.	Controls coat protein complex II (COP II) vesicles recruitment, ER‐Golgi transport, and cell surface delivery of some GPCRs.	[84]
PH4B	K69/114/130	Liver cancer cells: HepG2.	Stabilizes P4HB to mitigate oxidative stress and subsequent ER stress.	[101, 102]
HRD1	K610	Liver cancer cells: HepG2.	Prevents HRD1 from ubiquitination and degradation.	[103]
MEIS2	ND	Neuroblastoma (NB) cells: SK‐N‐BE (2) and SH‐SY5Y.	Promotes MEIS2 stability to maintain ER homeostasis and supports NB growth and progression.	[93]
VCP/p97	K109	Bone osteosarcoma epithelial cells: U2OS. Lung cancer cells: A549. Prostate cancer cells: DU145.	Promotes ATXN3‐mediated BECN1 deubiquitination and stability.	[88]
RPL26	K132/134	Colon cancer cells: HCT116. Bone osteosarcoma epithelial cells: U2OS. Erythroleukemia cells: K562.	Promotes ER‐phagy and degradation of the arrest peptides via lysosomes or proteasome system.	[73, 79, 89]
	K130/132/134/136/142	Liver cancer cells: HepG2. Hepatocellular carcinoma cells: Huh‐7.	Enhances Hepatitis A virus translation.	[76]
RPN1	ND	Colon cancer cells: HCT116.	Promotes ER‐phagy and suppresses IRE1α‐stimulated UPR.	[89]
CYB5R3	K214	Human cervical carcinoma cells: HeLa.	Converts CYB5R3 into its inactive form and signals macro‐ER‐phagy.	[92]
eIF4G1	K726/729	Bone osteosarcoma epithelial cells: U2OS. Human cervical carcinoma cells: HeLa.	Mediates eIF4F assembly and formation of the 48S pre‐initiation complex thereby promoting translation initiation.	[75]
Histone 4	K31	Bone osteosarcoma epithelial cells: U2OS. TNBC cells: MDA‐MB‐231.	Promotes the recruitment of Suv39H1 and Tip60 in DNA damage response.	[118]
MRE11	K282	Human cervical carcinoma cells: HeLa. Bone osteosarcoma epithelial cells: U2OS. Prostate cancer cells: DU145. Lung cancer cells: A549.	Promotes MRN complex assembly and subsequent enrichment at the DSB sites.	[120]
PARP1	K548	Human cervical carcinoma cells: HeLa. Lung cancer cells: A549.	Enhances PARP1 activity, thereby promoting CHK1 activation to maintain the stability and facilitate the restart of stalled replication fork.	[125]
PTIP	K148	Human cervical carcinoma cells: HeLa. Ovarian cancer cells: PEO‐1. TNBC cells: HCC1937. Pancreatic cancer cells: Capan‐1.	Facilitates assembly of the PTIP‐MLL3/4 complex, which enriches H3K4me1 and H3K3me3 and recruits MRE11 to stalled replication forks.	[122, 125]
p53	K351/357/370/373	Human cervical carcinoma cells: HeLa. Bone osteosarcoma epithelial cells: U2OS. Colon cancer cells: HCT116.	Inhibits MDM2‐mediated p53 proteasomal degradation.	[128]

Abbreviations: ND, no determination; TNBC, triple negative breast cancer.

### 3.1 UFMylation Is Indispensable for Protein Homeostasis

**FIGURE 2:**
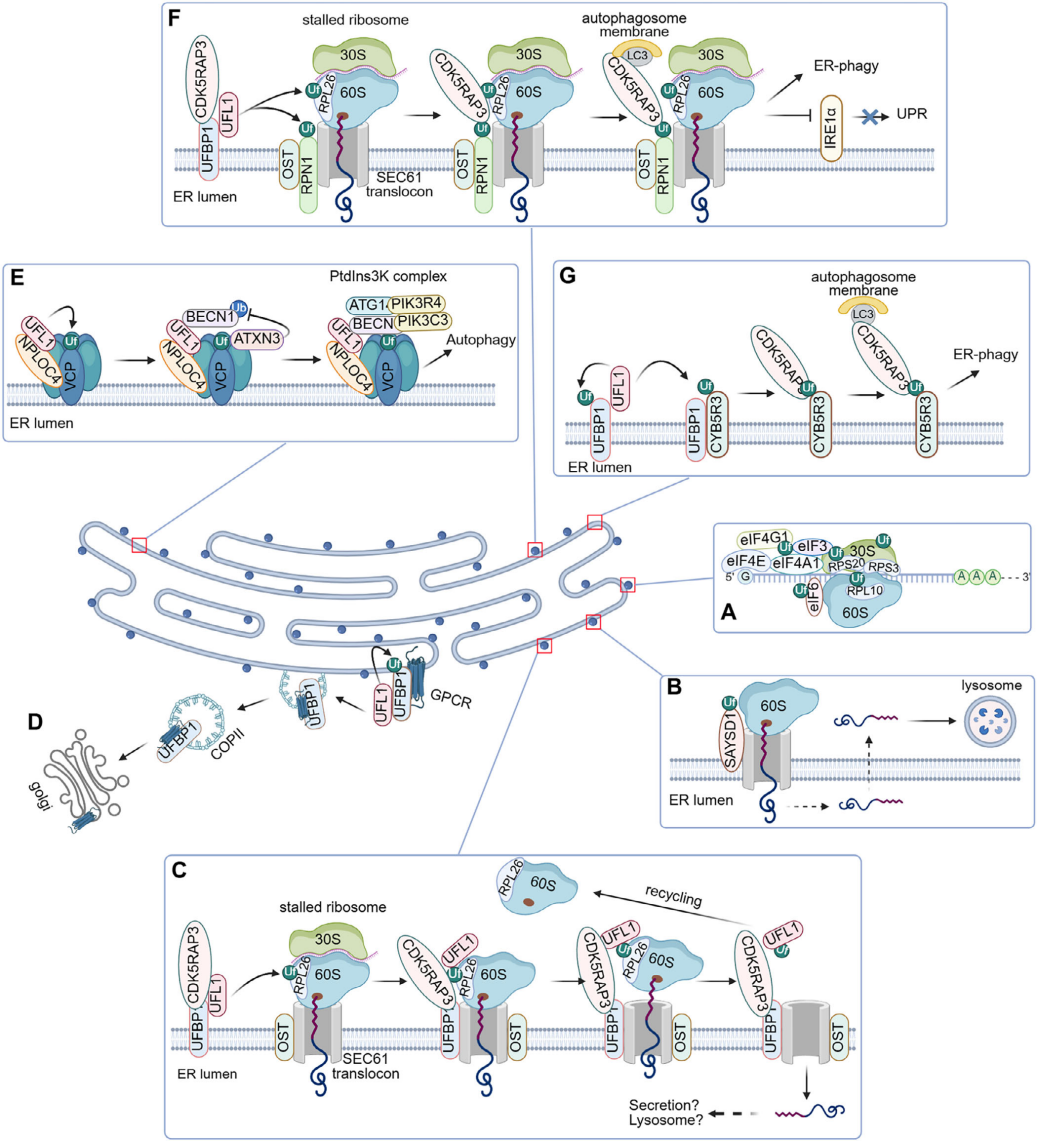
**UFMylation regulates protein homeostasis**, **sorting, autophagy**, **and ER‐phagy**. (A) Eukaryotic translation initiation factor 4G1 (eIF4G1) UFMylation facilitates eIF4E complex formation, and UFMylation of eIF6, ribosomal protein L10 (RPL10), RPL3, and ribosomal protein S20 (RPS20) enhances ribosome assembly and the interaction between mRNA and ribosome, thereby increasing translation. (B) Upon stress, translation mediated by ribosomes is stalled. Ribosome stalling induces RPL26 UFMylation which is indispensable for UFL1, UFBP1, CDK5RAP3, and UFM1 to form a C‐shaped clamp architecture that wraps around the 60S ribosomal subunit to obstruct the tRNA‐binding sites as well as the peptide exit tunnel, thereby dissociating the 60S subunit from the SEC61 translocon complex for recycling. The stalled, incomplete nascent peptides are degraded via lysosome. (C) Moreover, SAYSVFN motif domain‐containing 1 (SAYSD1) also recognizes UFMylated ribosomes to facilitate the clearance of translocation‐stalled proteins via lysosome. (D) UFMylation controls G‐protein‐coupled receptor (GPCR) sorting and delivery through coat protein complex II (COP II) vesicles. (E) With the assistance of nuclear protein localization protein 4 homolog (NPLOC4), valosin‐containing protein (VCP)/p97 is UFMylated. UFMylated VCP/p97 promotes the deubiquitination and stability of beclin‐1 (BECN1) via ataxin‐3 (ATXN3), thus promoting the formation of phosphatidylinositol 3‐kinase (PtdIns3K) complex. Ultimately, autophagy is induced. (F) Ribosome stalling induces the UFMylation of the 60S ribosome subunit RPL26 and the OST (oligosaccharyltransferase complex) complex subunit ribophorin‐1 (RPN1). CDK5RAP3 recognizes and binds UFMylated RPN1 and RPL26 and subsequently associates with LC3. This interaction enhances the association between the ER subdomain containing UFMylated RPL26 and the autophagosome membrane, thereby inducing ER‐phagy. (G) Simultaneously, IRE1α‐mediated UPR (unfolded protein response) is repressed. UFMylation of UFBP1 enhances the UFMylation of NADH‐cytochrome b5 reductase 3 (CYB5R3). CDK5RAP3 bridges the ER subdomain containing UFMylated CYB5R3 and the autophagosome membrane, leading to ER‐phagy.

Dysregulation of protein homeostasis usually leads to a variety of diseases, including cancers [70, 71]. UFMylation is deeply involved in protein homeostasis. A previous study indicates that the UFMylated ribosome‐associated proteins (RAPs) signal is exclusively enriched at fractions corresponding to the 60S subunit of ribosome in embryonic stem cells (ESCs) [72]. Further investigation shows that the RAP eIF6 (eukaryotic translation initiation factor 6), a key translation initiation factor that specifically associates with the 60S ribosome to prevent ribosomal subunit joining, is UFMylated (Figure 2). Moreover, three UFMylated ribosomal proteins (RPs), including two small subunit RPs, RPS3 and RPS20, as well as a large subunit RP RPL10, are also identified (Figure 2) [72]. RPS3, RPS20, and RPL10 are situated at the subunit interface, and RPS3 and RPS20 are located at close proximity to each other in the vicinity of the mRNA entrance [72]. These findings suggest that the UFMylation of RPS3, RPS20, RPL10, and eIF6 may synergistically control ribosome assembly and translation, and this prediction may be applied to cancer cells because the protein synthesis in both ESCs and cancer cells is aggravated. Additionally, UFMylation affects translation initiation complex assembly. UFMylation of eukaryotic translation initiation factor 4G1 (eIF4G1) at K729 and K726 facilitates the formation of eIF4E complex comprising eIF4G1, eIF4A1, eIF3, and eIF4E, thereby enhancing the translation of 50% proteins including most RPs and cell cycle protein CCND1 (Figure 2). Consequently, eIF4G1 UFMylation promotes proliferation of HeLa cells and U2OS cells [73].

RPL26 localizes on the large subunit of ribosomes tethered to the ER membrane and is adjacent to the polypeptide exit tunnel, the SEC61 translocon complex, and the oligosaccharyltransferase (OST) complex [74, 75]. Proteomic analysis indicates that UFMylation is indispensable for ER protein homeostasis in an ER‐associated degradation (ERAD)‐independent manner and RPL26 is UFMylated at K132 and K134 as the key target of UFMylation in K562 chronic myelogenous leukemia cells and U2OS osteosarcoma cells (Figure 2) [74]. Moreover, approximately 30% UFMylated RPL26 is associated with polysome, which actively translates mRNA into protein [74]. These results suggest that RPL26 UFMylation may be required for protein translation or co‐translational protein translocation into the ER. Unexpectedly, UFM1 knockout and UFSP2 knockout both have pretty tiny impact on translation, but adaptively change the transcriptome [74]. However, another research indicates that approximate 50% protein synthesis is blocked in UFC1‐depleted cells [73]. UFC1 depletion not only attenuates the translation of most RPs, including RPL26 and RPS3, but also disrupts the translation initiation complex by blocking the UFMylation of its core component eIF4G1, leading to cell cycle arrest in U2OS and HeLa cells [73]. Interestingly, RPL26 UFMylation is required for optimal translation of hepatitis A virus (HAV) RNA in host HepG2 and Huh‐7 hepatocellular carcinoma cells [76].

Ribosome stalling due to rare codons, specific mRNA structure, mRNA damage, or poor tRNA aminoacylation leads to accumulation of truncated peptides, which are deleterious for cells and must be eliminated by surveillance mechanisms, such as ribosome‐associated protein quality control (RQC) and ERAD [77, 78]. Ribosome stalling during protein translocation into the ER induces RPL26 UFMylation at K132 and K134, which facilitates the degradation of stalled incomplete nascent peptides (referred to as arrested peptides [APs]) through lysosome, but not RQC or ERAD, in K562 and HeLa cells (Figure 2) [79]. It is worth noting that another research group showed that UFMylated RPL26 collaborates with RQC machinery to degrade APs via lysosome or proteasome in U2OS cells [80]. Nevertheless, UFMylation safeguards the co‐translational protein translocation into the ER. Intriguingly, UFMylation triggers recycling of 60S ribosomal subunit from the ER in both normal cells and K562 cancer cells [81, 82]. Upon ribosome stalling or translation termination, RPL26 is UFMylated, and the UFMylation complex, including UFL1, UFBP1, CDK5RAP3, and UFM1, forms a C‐shaped clamp architecture that wraps around the 60S ribosomal subunit to obstruct the tRNA‐binding sites as well as the peptide exit tunnel, thereby dissociating the 60S subunit from the SEC61/SEC62/SEC63 translocon complex for recycling. Importantly, the UFMylation complex also functions as a potential UFMylation “reader” in this process (Figure 2) [81, 82]. SAYSVFN motif domain containing 1 (SAYSD1) is another putative “reader” of UFMylation because it recognizes UFMylated ribosome to facilitate the clearance of translocation‐stalled proteins and consequently ensures ER protein homeostasis (Figure 2) [83]. Unexpectedly, UFMylation participates in protein sorting at the ER. UFL1 and UFBP1 control coat protein complex II (COP II) vesicles recruitment, ER‐Golgi transport, and cell surface delivery of some GPCRs in a UFMylation‐independent fashion in HeLa cells and HT29 colorectal adenocarcinoma cells (Figure 2) [84].

### 3.2 UFMylation Is Involved in Autophagy and ER‐phagy

Autophagy is an intracellular degradation system that controls organelle mass and functions by lysosomal/vacuolar degradation of the excess or defective portions of organelles [85, 86]. ER‐phagy is a selective autophagy that specifically degrades the subdomains of the ER within lysosomes/vacuoles [85, 86]. Several key ER‐phagy or autophagy‐associated proteins are engaged in tumor growth and progression, such as valosin‐containing protein (VCP)/p97, FAM134B, SEC63, CALCOCO1, and TEX264 [86, 87]. However, the regulatory mechanisms for ER‐phagy and autophagy are not well understood.

VCP/p97 is essential for autophagy initiation. Recent research has demonstrated that UFMylation is important for VCP/p97‐mediated autophagy in cancer cells, such as U2OS osteosarcoma cells, A549 cells, and DU145 prostate cancer cells. Various VCP/p97 mutations have been identified in multiple cancers, suggesting its potential roles in tumorigenesis [88]. NPLOC4 facilitates the binding of VCP/p97 to UFL1 and promotes VCP/p97 UFMylation on K109. VCP/p97 UFMylation promotes ATXN3‐mediated BECN1 deubiquitination and stability. As a core component of the PtdIns3K complex, stabilized BECN1 augments PtdIns3K complex assembly (Figure 2). Hence, VCP/p97 UFMylation is indispensable for autophagy in cancer cells [88].

A genome‐wide ER‐phagy screen identified UFMylated RPL26 and ribophorin 1 (RPN1, a subunit of the OST complex) as positive regulators of ER‐phagy, which suppresses IRE1α‐stimulated UPR [89]. UFL1 is recruited by the ER surface adaptor UFBP1 to the ER and UFMylates ER surface proteins RPL26 and RPN1 that are in close proximity. CDK5RAP3 associates with UFMylated RPL26 and RPN1 in the ER subdomains, as well as LC3 in the autophagosome membrane. Therefore, the interaction between the ER subdomains and the autophagosome membrane is enhanced, leading to autophagy of ER sheets (Figure 2) [89, 90]. Notably, the interaction between UFL1 and UFBP1, as well as UFL1‐mediated UFBP1 stability, is not affected by UFM1 knockdown. This suggests that UFBP1 is not a target of UFMylation during ER‐phagy, although UFBP1 has been reported to be UFMylated in the UPR and other pathways [89].

NADH‐cytochrome b5 reductase 3 (CYB5R3) is an ER‐localized reductase anchored to the ER by an N‐terminal long hydrophobic stretch, with FAD‐and NADH‐binding domains contributing to fatty acid and cholesterol biogenesis [91]. CYB5R3 is UFMylated by UFL1 and UFBP1 at K214, situated between the NADH and FAD domains. When CYB5R3 undergoes UFMylation, its molecular conformation shifts towards a closed state, disrupting the FAD‐binding groove and inactivating CYB5R3. UFMylated CYB5R3 is recognized by UFBP1, further enhancing CYB5R3 UFMylation. Thereafter, CDK5RAP3 enhances the interaction between the ER subdomains and the autophagosome membrane through interacting with UFMylated CYB5R3 and LC3. Ultimately, ER‐phagy is induced and UFMylated CYB5R3 is degraded by ER‐phagy in HeLa cells (Figure 2) [92]. However, CYB5R3 UFMylation barely influences the synthesis of fatty acid or cholesterol, probably due to compensatory effect of other metabolic enzymes [92]. In addition, CDK5RAP3 dysfunction triggers ER‐phagy in NB cells [93]. Collectively, UFMylation controls ER‐phagy via different mechanisms in cancer cells.

### 3.3 UFMylation Modulates the UPR Signaling Pathways

The homeostasis of redox, calcium, and glucose is frequently disturbed in cancer cells, leading to ER stress and activation of the unfolded protein response (UPR) [94, 95]. Cancer cells exploit the UPR to manage ER stress and promote survival, although UPR hyperactivation can lead to maladaptive cell death [96, 97]. The UPR consists of three main signaling pathways: inositol‐requiring enzyme 1α (IRE1α), protein kinase R‐like ER resident kinase (PERK), and activating transcription factor 6 (ATF6), which collectively enable cancer cells to cope with hostile environmental conditions [98]. It is well established that the UFMylation system is indispensable for maintaining ER homeostasis [17, 18], indicating its critical role in modulating UPR pathways in cancer cells.

**FIGURE 3:**
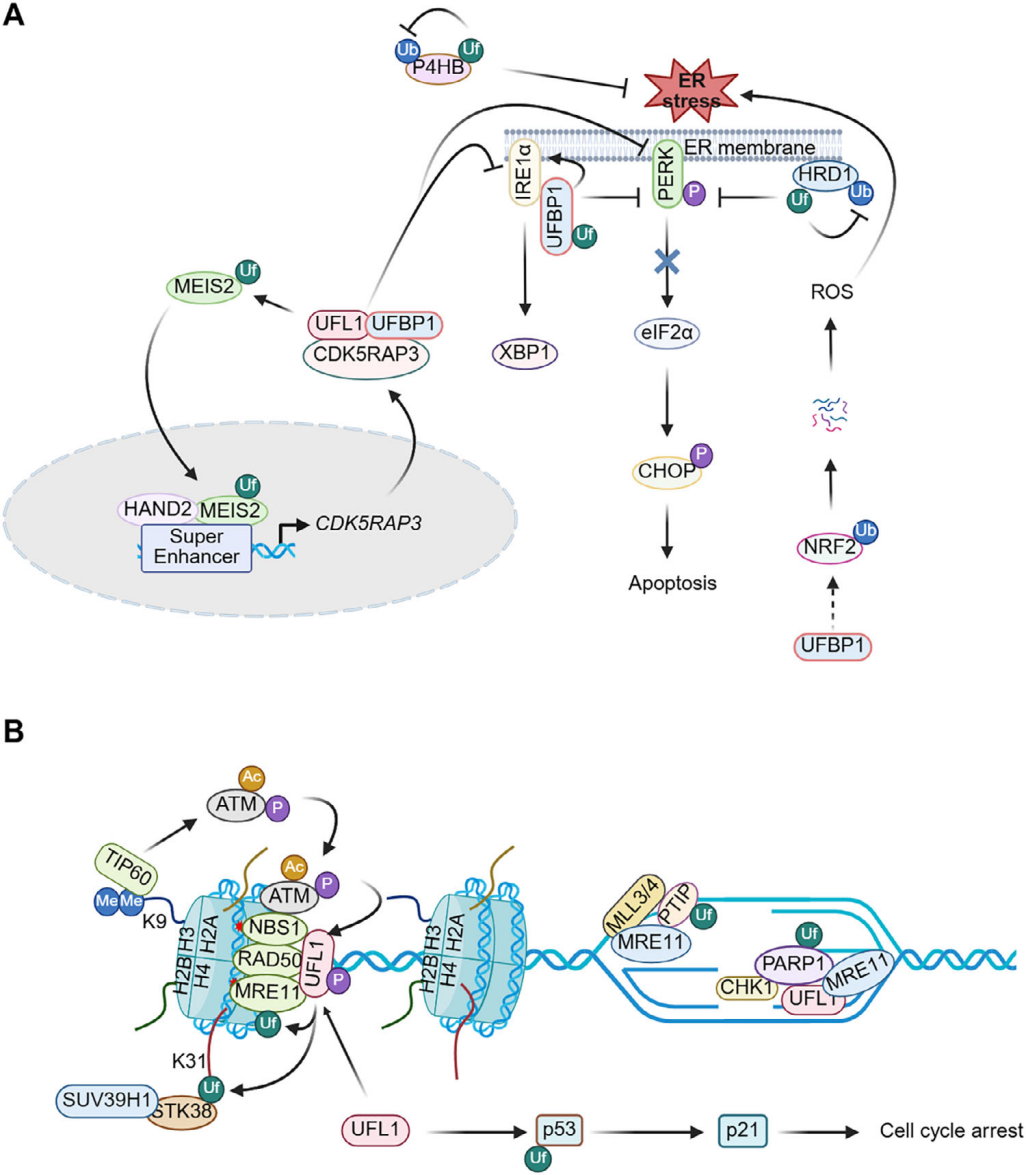
**UFMylation regulates UPR and genome stability maintenance**. (**A**) The UFL1‐CDK5RAP3‐UFBP1 complex triggers MEIS2 UFMylation, enhancing its stability and nuclear translocation to promote CDK5RAP3 transcription while suppressing inositol‐requiring protein 1α (IRE1α)‐ and pancreatic eIF2‐alpha kinase (PERK)‐mediated UPR. UFMylated UFBP1 stabilizes unphosphorylated IRE1α, activating the IRE1α‐XBP1s (spliced X‐box‐binding protein 1) axis while suppressing the PERK‐eIF2α‐CHOP (C/EBP‐homologous protein) signaling pathway. In addition, the UFMylation of HRD1 inhibits its ubiquitylation and degradation, thereby suppressing the PERK‐eIF2α‐CHOP signaling pathway. Moreover, UFMylation attenuates UPR by inhibiting the ubiquitination and degradation of the ER stress inhibitor prolyl 4‐hydroxylase subunit beta (P4HB). In contrast, UFBP1 promotes reactive oxygen species (ROS) generation by enhancing nuclear factor erythroid 2‐related factor 2 (NRF2) ubiquitination and degradation, resulting in ER stress and UPR. (**B**) During DNA double‐strand breaks (DSB) repair, UFL1‐mediated UFMylation of MRE11 and histone H4 facilitates MRN (MRE11‐RAD50‐NBS1) complex assembly and serine/threonine‐protein kinase 38 (STK38)‐dependent recruitment of suppressor of variegation 3‐9 homolog 1 (SUV39H1), respectively. SUV39H1‐mediated H3K9 methylation recruits 60 kDa Tat‐interactive protein (TIP60) to acetylate and activate ATM, while ATM‐phosphorylated UFL1 creates a positive feedback loop. Under replication stress, UFMylation of PARP1 and PAX transactivation activation domain‐interacting protein (PTIP) at stalled forks enhances: (i) PARP1's PARylation activity to promote MRE11‐mediated DNA resection and checkpoint kinase‐1 (CHK1)‐dependent cell cycle arrest, and (ii) PTIP‐MLL3/4 complex formation to recruit MRE11 for resection.

UFBP1 specifically associates with and stabilizes unphosphorylated IRE1α while attenuating PERK phosphorylation in MCF‐7 breast cancer cells and HepG2 liver cancer cells (Figure 3A) [99]. This selective stabilization leads to activation of the IRE1α‐XBP1s axis and suppression of the PERK‐eIF2α‐CHOP signaling pathway, thereby preventing apoptosis in these cancer cells. Interestingly, UFMylation of UFBP1 at K267 is a prerequisite for its role in regulating UPR signaling pathways (Figure 3A) [99]. These findings can be extended to hematopoietic stem cells (HSCs), which are extremely sensitive to ER stress [99, 100]. Additionally, UFL1 UFMylates prolyl 4‐hydroxylase beta (P4HB) at K69, K114, and K130, stabilizing P4HB, an inhibitory factor of ER stress. This process mitigates oxidative stress and subsequent ER stress in HepG2 cells (Figure 3A) [101, 102]. In gastric cancer cells, UFL1 and UFBP1 collaboratively UFMylate the multi‐transmembrane protein HRD1 at K610, inhibiting its ubiquitination and degradation. Consequently, this stabilizes HRD1 and decreases the protein levels of IRE1α, PERK, phosphorylated PERK (p‐PERK), and XBP1s, thereby suppressing UPR and apoptosis in cancer cell upon ER stress (Figure 3A) [103]. In neuroblastoma (NB) cells, the transcriptional factor Meis homeobox 2 (MEIS2) enhances CDK5RAPS expression by binding to its super enhancer (Figure 3A) [93]. CDK5RAP3 then facilitates the assembly of the UFL1‐CDK5RAP3‐UFBP1 complex, promoting MEIS2 UFMylation and stability (Figure 3A). This process downregulates the IRE1α and PERK pathways, maintaining ER homeostasis and supporting NB growth and progression through a positive feedback loop (Figure 3A) [93]. Furthermore, UFMylation enhances ER‐phagy to suppress IRE1α‐mediated UPR in HCT116 colon cancer cells [89]. Together, these findings indicate that UFMylation attenuates UPR signaling to prevent apoptosis and sustain tumor growth and progression in various cancers.

In contrast to the pro‐survival role of UFMylation in some cancer cells, UFMylation can stimulate apoptosis by attenuating the IRE1α pathway in lung cancer cells [104]. The ablation of UFM1, UFC1, or UBA5 significantly increases the protein levels of p‐IRE1α without affecting the protein levels of total IRE1α, total PERK, p‐PERK, or eIF2α. This triggers a protective UPR and enhances the importance of the anti‐apoptotic effector Bcl‐xL for the survival of TKI‐tolerant cells in the absence of UFMylation [104]. Interestingly, the multi‐targeted TKI sunitinib dramatically inhibits UFMylation, whereas high UFMylation activity increases the resistance of renal cancer cells to sunitinib [105]. ER stress induces the nuclear translocation of the transcription factor NRF2, which eliminates ER stress‐induced excessive reactive oxygen species (ROS) and other oxidants, thereby suppressing apoptosis [106]. In gastric cancer cells, UFBP1 interacts with NRF2 and promotes its ubiquitination and proteasomal degradation, suggesting that NRF2 UFMylation may upregulate ROS levels and lead to UPR‐induced apoptosis (Figure 3A) [107]. From this perspective, UFMylation may provoke UPR‐induced apoptosis in lung cancer cells and gastric cancer cells by modulating the IRE1α pathway and NRF2 degradation.

### 3.4 UFMylation Fine‐Tunes Genome Stability

Genome instability caused by DNA damage is considered a major etiological factor as well as a hallmark of tumorigenesis [108, 109]. To avoid severe DNA damage, the DNA damage response (DDR) pathways, including the mismatch (MMR) repair pathway for replicative errors or mismatch, the base‐excision repair (BER) pathway for single‐strand DNA breaks (SSB), and the non‐homologous end joining (NHEJ), and homologous recombination (HR) repair pathways for double‐strand breaks (DSB), are immediately activated when DNA damage occurs [110]. Defective and hyperactive DDR both lead to genome instability [111]. Therefore, the DDR must be tightly controlled to maintain genome stability.

It has long been reported that the components of the UFMylation system, including CDK5RAP3 and UFL1, are involved in DNA damage repair. CDK5RAP3 overcomes the G2/M DNA damage checkpoint to sensitize cancer cells to NDA‐damaging reagents by activating CDK1 [112, 113]. Moreover, CDK5RAP3 interacts with and enhances the intrinsic catalytic activity of wild‐type p53‐induced phosphatase 1 (WIP1), which is a negative regulator of DDR, indicating that CDK5RAP3 may suppress DDR via WIP1 [114, 115]. However, these findings did not establish a relationship between DDR and UFMylation because CDK5RAP3 had not been identified as a component of the UFMylation system until 2022. In 2015, UFL1 depletion‐induced DNA damage was observed in HCT116 [116], although the UFMylation‐independent function of UFL1 cannot be ruled out in this phenomenon. The breakthrough occurred in 2019, when two independent groups established a direct connection between DDR and UFMylation.

The kinase ataxia‐telangiectasia mutated (ATM) and its upstream regulator MRN (MRE11‐RAD50‐NBS1) complex is critical for DSB repair [117]. However, the mechanism of ATM activity regulation by the MRN complex remains largely unknown. Lou's group demonstrates that the MRN complex hijacks UFL1‐mediated H4 UFMylation to activate ATM in breast cancer cells and osteosarcoma cells [118]. When DSB occurs, UFL1 is recruited to DBS sites by the MRN complex to mono‐UFMylate histone 4 (H4) at K31 (Figure 3B). Methyltransferase suppressor of variegation 3‐9 homolog 1 (SUV39H1) recognizes and binds to UFMylated H4 to catalyze the formation of H3K9me3, which provides a platform for TIP60 to acetylate and consequently activate ATM (Figure 3B). In turn, activated ATM phosphorylates UFL1 at S462 to reinforce its activity (Figure 3B). Therefore, UFL1 and ATM form a positive feedback loop to repair DSB [118]. Furthermore, the serine/threonine kinase 38 (STK38) is demonstrated to be indispensable for the recruitment of SUV39H1 to UFMylated H4 at DSB sites (Figure 3B) [119]. UFL1 also controls DSB repair by directly UFMylating MRE11 in U2OS, A549, and DU145 cancer cells, contributing to the complexity of UFMylation‐mediated DSB repair [120]. Upon DSB, UFL1 associates with and UFMylates MRE11 at K282, thereby promoting MRN complex assembly and subsequent enrichment at the DSB sites (Figure 3B) [120]. MRE11 UFMylation by UFL1 is confirmed in HeLa cells, BRCA2‐deficient cancer cells, and zebrafish, and it is critical for telomere length maintenance and nascent DNA degradation [121, 122]. Unexpectedly, UFL1 depletion in HeLa cells and BRCA2‐deficient cancer cells does not affect DSB repair or MRN complex formation [121, 122], and the interaction between MRE11 and components of the UFMylation system is not impacted by DSB [121].

Replication stress, an impeded replication fork progression, has emerged as a significant cause of genome instability in cancer cells [123]. Therefore, maintaining the stability and facilitating the restart of stalled replication forks are critical for preventing DNA damage. Given the pivotal roles of UFMylation in the DDR, it is plausible that the UFMylation system participates in the regulation of replication forks. Indeed, UFL1 modulates replication fork stability by UFMylating PARP1 and PTIP in human cancer cells [122, 124, 125]. In BRCA2‐proficient cancer cells exposed to replication stress, UFL1 UFMylates PARP1 at K548, thereby enhancing its PARylation activity. Activated PARP1 promotes CHK1 activation, leading to cell cycle arrest and facilitating MRE11‐mediated nascent DNA resection and stalled replication fork restart (Figure 3B). Conversely, in BRCA2‐deficient cancer cells, PARP1 UFMylation results in excessive resection of replication forks by MRE11, resulting in replication fork collapse and triggering genome instability (Figure 3B) [124]. In addition to the UFL1‐PARP1‐MRE11/CHK1 axis, the UFL1‐PTIP‐MRE11 signaling also plays a critical role in the regulation of replication fork [122, 125]. In BRCA1/2‐deficient cancer cells under replication stress, UFL1 localizes to stalled replication forks and UFMylates PTIP at K148 (Figure 3B). This modification facilitates assembly of the PTIP‐MLL3/4 complex, which enriches H3K4me1 and H3K4me3 and recruits MRE11 to stalled replication forks (Figure 3B). Consequently, MRE11 extensively degrades nascent DNA at these forks, thereby reducing genome stability in BRCA1/2‐negative cancer cells. These findings highlight the collaborative role of UFMylated PARP1 and PTIP in ensuring genome stability in BRCA1/2‐proficient cancer cells while inducing genome instability in BRCA1/2‐deficient cancer cells.

The transcription factor p53 plays a critical role in safeguarding genome stability through various mechanisms. Upon DNA damage, ATM and ATR (ATM and Rad3‐related) kinases phosphorylate and activate p53 via CHK2 and CHK1, respectively, leading to the expression of the cell cycle inhibitor p21. This activation halts the cell cycle, allowing DNA repair machineries to eliminate DNA damage [126, 127]. Additionally, p53 can directly regulate multiple DDR pathways, including MMR, BER, HR, and NHEJ [127]. Despite its crucial role, wild‐type p53 has a short lifespan under normal conditions and is frequently mutated in a plethora of human cancers. Mutant p53 loses the capacity to induce cell cycle arrest, senescence, or apoptosis in response to DNA damage, allowing cancer cells to proliferate and accumulate mutations that promote tumor progression [126]. Recent studies have highlighted the role of UFMylation in regulating p53 accumulation in response to DNA damage [128]. UFL1 competes with MDM2 by interacting directly with and UFMylating p53 at K351, K357, K370, and K373 (Figure 3B). This UFMylation inhibits MDM2‐mediated p53 proteasomal degradation in various cancer cells, including HeLa, U2OS, and HCT116 cells (Figure 3B) [128]. Interestingly, decreased levels of UFL1 and UFBP1 correlate positively with p53 levels in renal cell carcinoma (RCC) [128]. Further investigation is needed to elucidate how DNA damage triggers p53 UFMylation.

### 3.5 UFMylation Is Critical for Tumor Immunity

T cells selectively surveil and eliminate pathogens and abnormal cells, including cancer cells, to maintain the body's homeostasis [129]. However, hyperactivated T cells may also attack normal cells, leading to autoimmune diseases [130]. To avoid autoimmune reactions, multiple negative regulatory checkpoint pathways have evolved to maintain optimal T‐cell activity. Among these, the programmed death‐1 (PD‐1, also known as PDCD1 or CD279)/programmed death ligand‐1 (PD‐L1) pathway is the most intensively studied [131]. However, cancer cells often hijack the PD‐1/PD‐L1 pathway to induce immunosuppression in the tumor microenvironment [131, 132]. Recent studies have identified UFMylation as a crucial regulator of the PD‐1/PD‐L1 pathway, although findings in this area are contradictory.

**FIGURE 4:**
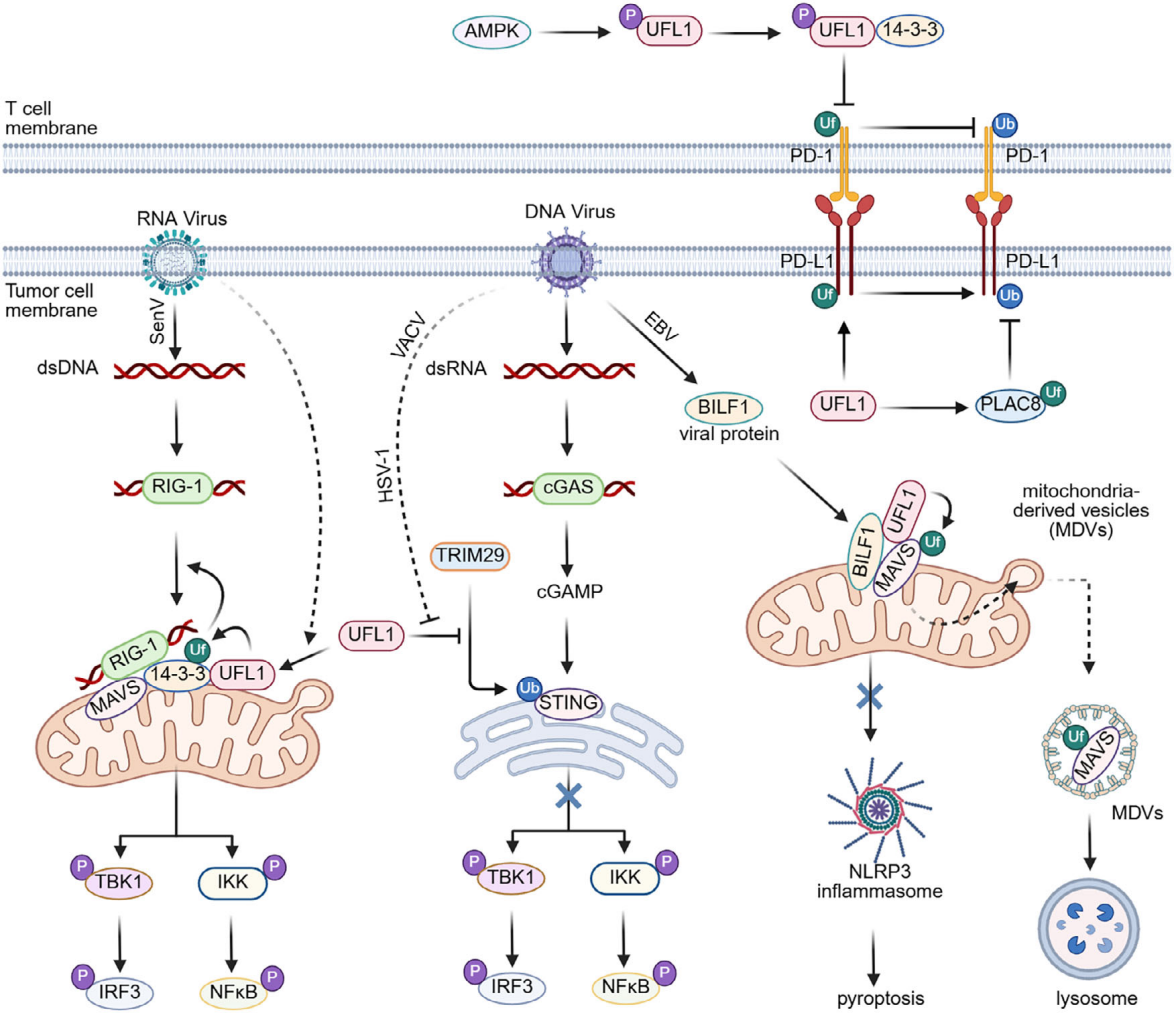
**Impact of the UFMylation system on the immune response**. 5'‐AMP‐activated protein kinase (AMPK) phosphorylates UFL1, strengthening the UFL1‐14‐3‐3 interaction, which suppresses programmed cell death protein 1 (PD‐1) UFMylation and subsequent destabilization on the T‐cell membrane, thereby abolishing PD‐1‐mediated immunosuppression. UFMylation of programmed cell death 1 ligand 1 (PD‐L1) destabilizes it by enhancing its ubiquitination and proteasomal degradation, whereas UFMylated placenta‐specific gene 8 protein (PLAC8) increases PD‐L1 protein levels by preventing its ubiquitination and degradation. RNA virus infections induce UFL1‐mediated 14‐3‐3ε UFMylation, enhancing retinoic acid‐inducible gene 1 (RIG‐I)‐mitochondrial antiviral‐signaling protein (MAVS) innate immune signaling. Consequently, the interferon regulatory factor 3 (IRF3) and nuclear factor kappa‐light‐chain‐enhancer of activated B cells (NF‐κB) signaling pathways are stimulated to produce type I IFN and inflammatory factors. DNA viruses promote TRIM29‐mediated STING ubiquitination and degradation by decreasing UFL1 protein levels. Destabilized STING abolishes the expression of type I interferon (IFN) and inflammatory factors. Additionally, the DNA virus protein BamHI‐I leftward reading frame 1 (BILF1) recruits UFL1 to mediate MAVS UFMylation, which leads to the selective removal of MAVS from the mitochondrial outer membrane and its packaging into mitochondria‐derived vesicles (MDVs) for lysosomal degradation, thereby enhancing cancer cell survival by blocking MAVS‐mediated NACHT, LRR, and PYD domains‐containing protein 3 (NLRP3) inflammasome activation and subsequent pyroptosis. Dash lines indicate unresolved mechanisms, and solid lines present known mechanisms.

Placenta‐specific 8 (PLAC8) UFMylation suppresses its degradation through the lysosome, thereby increasing PLAC8 stability in triple‐negative breast cancer (TNBC) cells, including MDA‐MB‐231. Consequently, PLAC8 upregulates PD‐L1 protein levels by inhibiting PD‐L1 ubiquitination and degradation. Ultimately, UFMylated PLAC8 attenuates T lymphocyte activity, promoting TNBC growth (Figure 4) [133]. These findings corroborate earlier studies identifying PLAC8 as an oncogenic factor in breast cancer [134]. Collectively, UFMylated PLAC8 exerts an immunosuppressive role by increasing PD‐L1 expression in TNBC. Thus, the UFMylation system appears to possess oncogenic potential in TNBC. In contrast, conflicting research shows that the UFMylation system acts as a tumor suppressor by promoting proteasomal degradation of PD‐L1 in TNBC, liver cancer, and melanoma cells [135]. PD‐L1 undergoes UFMylation at K75, K89, K105, K162, K280, and K281, facilitating its ubiquitination and degradation. This process suppresses the interaction between MDA‐MB‐231 breast cancer cells and T cells, thereby inhibiting T‐cell‐mediated antitumor immunity (Figure 4). The effect of UFMylation on PD‐L1 has yielded opposing conclusions across different research groups, even within the same cell lines, potentially attributable to variations in lysis buffer composition or experimental techniques. Notably, the protein levels of the core components and the clinical relevance of the UFMylation system in TNBC have not been thoroughly analyzed in these studies.

Recently, UFL1 has been demonstrated to suppress T‐cell activation and anti‐tumor immunity in UFL1 conditional knockout (cKO) mice. UFL1 promotes PD‐1 UFMylation at K210 and K233, major sites that counteract K48‐linked ubiquitination and proteasomal degradation of PD‐1 in T cells, thereby inhibiting T‐cell activation and leading to reduced tumor growth (Figure 4) [136]. Importantly, UFL1 has no impact on PD‐L1 in T cells, indicating distinct substrates for UFMylation in tumor cells and T cells [136]. Furthermore, UFL1‐mediated anti‐tumor immunosuppression is regulated by energy status. AMP‐activated protein kinase (AMPK), activated under low energy conditions, phosphorylates UFL1 at T536, enhancing the UFL1‐14‐3‐3 interaction (Figure 4). This interaction reduces UFL1‐PD‐1 binding, PD‐1 UFMylation, and subsequently destabilizes PD‐1. PD‐1 destabilization promotes T‐cell activation and enhances anti‐tumor immunity (Figure 4) [136].

The DNA virus Epstein–Barr virus (EBV) contributes to approximately 2% of all human cancers, including lymphoma and gastric carcinoma [137, 138]. EBV has been reported to dismantle the NLRP3 inflammasome by mediating UFMylation‐mediated MAVS degradation, ensuring the survival of the gastric cancer cells and lymphoma cells. EBV‐encoded G‐protein‐coupled receptor (GPCR) BILF1 is located on the outer membrane of host cell mitochondria, where it facilitates MAVS UFMylation at K461 by UFL1. UFMylated MAVS then recruits the E3 ligase PARK2 for ubiquitination and selective packaging into mitochondrial‐derived vesicles (MDVs) (Figure 4). Subsequently, these MDVs are degraded in lysosomes. As a result, MAVS‐induced NLRP3 inflammasome activation and subsequent pyroptosis are suppressed to enhance the survival of gastric carcinoma cells and lymphoma cells (Figure 4). Moreover, DNA virus herpes simplex virus (HSV‐1) and vaccinia virus (VACV) infections both downregulate the protein levels of UFL1, which competes with E3 ligase TRIM29 to bind STING, leading to elevated STING ubiquitination and degradation independent of UFMylation. Destabilized STING alleviates IRF3‐and NF‐κB‐mediated expression of type I IFN and inflammatory cytokines to attenuate antiviral immune response in A549 lung cancer cells and HeLa cells (Figure 4) [139]. In contrast, Sendai RNA virus (SenV) infections enhance the interaction between UFL1 and 14‐3‐3ε leading to 14‐3‐3ε UFMylation and promotion of the RIG‐I‐MAVS innate immune signaling (Figure 4) [140]. It is worth noting that in tyrosine kinase inhibitor (TKI)‐tolerant lung cancer cells, UFM1 ablation increases the protein levels of STING, a MAVS counterpart, activating pro‐survival NF‐κB signaling and thereby preventing apoptosis in lung cancer cells [104].

### 3.6 UFMylation Regulates Estrogen‐Related Transcription

**FIGURE 5:**
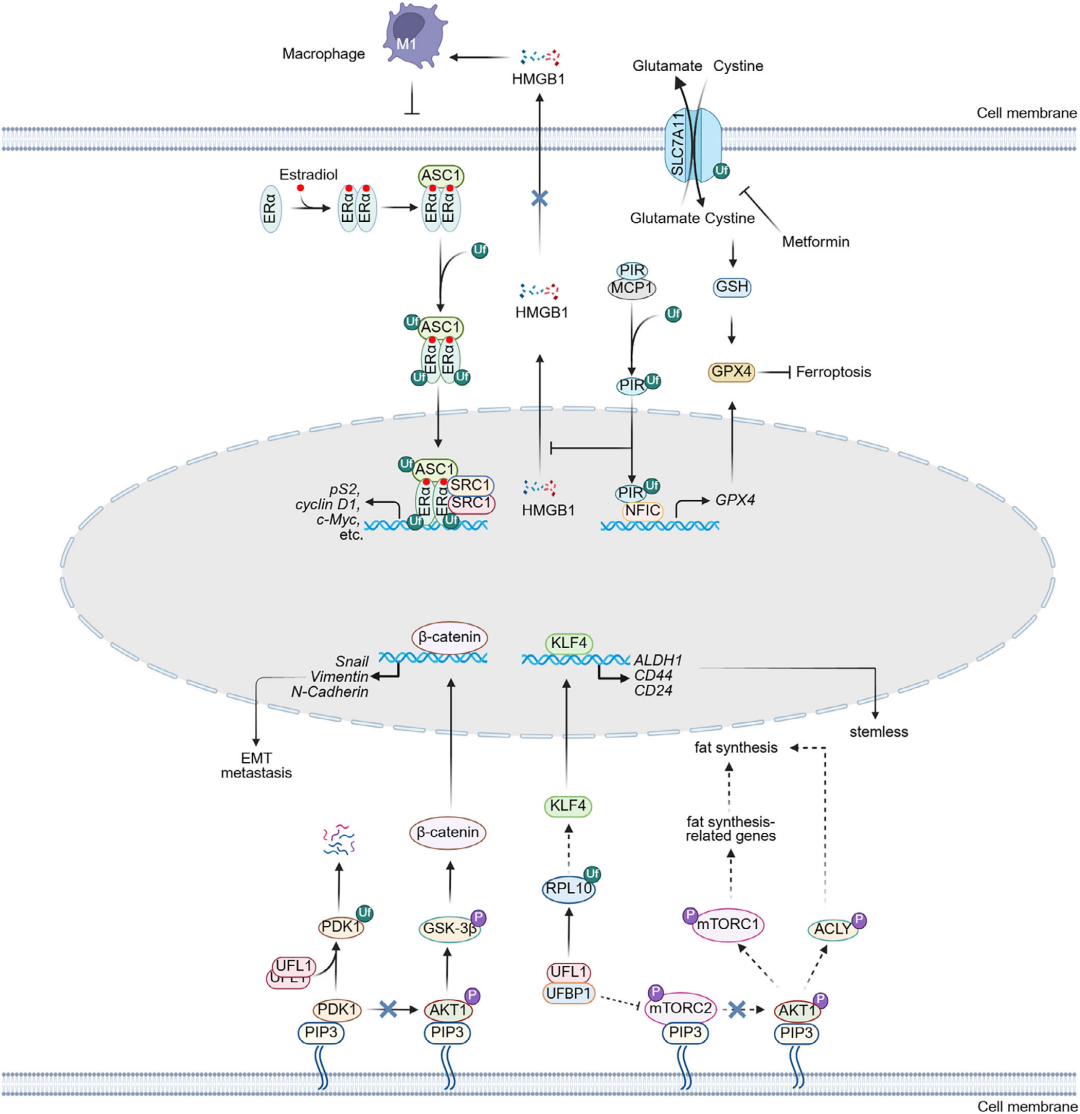
**UFMylation regulates ERα signaling, ferroptosis, pluripotency, metastasis, and lipid metabolism**. In the presence of 17β‐estradiol, ERα associates with asc‐type amino acid transporter 1 (ASC1), leading to ASC1 UFMylation and providing a platform for the enrichment of SRC1 and p300 on estrogen‐responsive elements (EREs) of ERα target genes. In addition, ERα is UFMylated and stabilized. Ultimately, the UFMylation of ASC1 and ERα promotes estrogen receptor‐positive (ER^+^) breast tumor growth by provoking the transcription of ERα target genes. Solute carrier family 7 member 11 (SLC7A11) UFMylation increases its stability, cystine import, and glutathione (GSH) synthesis, thereby promoting glutathione peroxidase 4 (GPX4) activity and inhibiting ferroptosis. Moreover, macrophage‐capping protein 1 (MCP1) enhances pirin (PIR) UFMylation and stability. Stabilized PIR interacts and cooperates with the transcriptional factor nuclear factor I‐C (NFIC) to stimulate GPX4 transcription, leading to decreased ferroptosis. On the other hand, PIR inactivates antitumor pro‐inflammatory M1‐like macrophages by blocking the cytoplasmic transport of high mobility group box 1 (HMGB1). UFMylation of PDK1 increases its ubiquitination and degradation, resulting in reduced AKT1 and glycogen synthase kinase‐3 beta (GSK‐3β) activities. Consequently, the expression of β‐catenin‐induced epithelial‐to‐mesenchymal transition (EMT) genes is decreased and cancer cell metastasis is alleviated. UFL1 and UFBP1 may suppress lipogenesis by abolishing mTORC2 phosphorylation in cancer cells. Conversely, UFMylation of RPL10 results in elevated levels of the pluripotency factor krueppel‐like factor 4 (KLF4) and its downstream target genes, maintaining cancer cell stemness.

In hormone‐related cancers, estrogen receptor‐α (ERα) is a well‐known growth factor that promotes ERα‐positive breast tumor growth [141, 142, 143, 144, 145]. Upon 17β‐estradiol stimulation, ERα forms a homodimer and binds to the ER‐responsive elements (EREs) of ERα target genes, then recruits transcriptional coactivators, including SRC1 and p300, to induce the transcription of ERα target genes [146, 147, 148]. However, the mechanism by which SRC1 and p300 are recruited by ERα remains elusive. Yoo et al. demonstrated that UFMylated activating signal cointegrator 1 (ASC1) is essential for ERα transactivation [149]. In the absence of 17β‐estradiol, ASC1 is maintained in a non‐UFMylated state by UFSP2. Upon 17β‐estradiol binding, ERα competes with UFSP2 to associate with ASC1, leading to ASC1 UFMylation at K324, K325, K334, and K367 by UFL1 and UFBP1 (Figure 5). UFMylated ASC1 provides a platform for SRC1 and p300 enrichment at the EREs of ERα target genes (Figure 5). Ultimately, UFMylated ASC1 promotes ERα‐mediated breast tumor growth (Figure 5) [149]. Intriguingly, ERα is also UFMylated at K171 and K180 to prevent it from ubiquitination and subsequent degradation (Figure 5) [150]. Importantly, the protein levels of UFM1, UBA5, UFC1, UFL1, and UFBP1 are all upregulated in ERα^+^ human breast tissues. Collectively, UFMylation promotes ERα^+^ breast tumor growth by simultaneously enhancing ERα stability and facilitating the assembly of the ERα‐ASC1‐P300‐SRC1 transcriptional complex.

### 3.7 UFMylation Controls Cancer Cell Ferroptosis

Ferroptosis is an iron‐dependent form of cell death that has emerged as a prominent area of research interest [151, 152, 153, 154]. Ferroptosis occurs when membrane lipid peroxides accumulate to toxic levels [155, 156, 157, 158, 159, 160, 161]. Despite significant advances in ferroptosis research, the mechanism of ferroptosis regulation remains poorly understood. Yang et al. showed that UFMylation of solute carrier family 7 member 11 (SLC7A11), a molecule implicated in the glutamate‐cystine antiporter and glutathione (GSH) synthesis, increases SLC7A11 stability, thereby suppressing lipid peroxides and subsequent ferroptosis (Figure 5). Consequently, SLC7A11 UFMylation accelerates ERα^+^ breast tumor growth. Interestingly, metformin downregulates UFM1 expression to decrease SLC7A11 UFMylation. Thus, metformin inhibits ERα‐positive breast tumor growth by diminishing SLC7A11 UFMylation (Figure 5) [162]. Intriguingly, UFL1 also inhibits lipopolysaccharide (LPS)‐induced ferroptosis in human granulosa‐like cells (KGNs) by upregulating SLC7A11 expression [163], although the underlying mechanism has not yet been deciphered.

Coincidentally, UFMylation of pirin (PIR) impedes ferroptosis in pancreatic ductal adenocarcinoma (PDAC) (Figure 5) [164]. Mechanistically, macrophage‐capping protein (MCP) facilitates pirin (PIR) UFMylation at K5 and K6, thereby preventing its proteasomal degradation. Stabilized PIR interacts and cooperates with the transcription factor nuclear factor I‐C (NFIC) to stimulate GPX4 transcription, leading to decreased lipid peroxidation and ferroptosis (Figure 5) [164]. Concurrently, PIR inactivates antitumor pro‐inflammatory M1‐like macrophages by blocking the cytoplasmic transport of high mobility group box 1 (HMGB1) (Figure 5) [164]. Collectively, PIR presents an attractive therapeutic target for PDAC due to its dual pro‐tumor effect.

### 3.8 UFMylation Contributes to Cancer Cell Stemness and Metastasis

RPs play crucial roles in cancer cell proliferation by facilitating ribosome assembly and protein synthesis [165]. Ribosomal protein L10 (RPL10) UFMylation is implicated in maintaining pluripotency in pancreatic adenocarcinoma (PAAD) stem cells [166]. UFM1 is conjugated to K30 and K101 on RPL10, which leads to increased expression of the pluripotency factor kruppel‐like factor 4 (KLF4) and its downstream targets, including aldehyde dehydrogenase 1 (ALDH1), CD44, and CD24‐well‐known markers of PAAD stem cells (Figure 5). Thus, the UFMylation system enhances the stemness of cancer cells. This finding is supported by research in glioblastoma stem cells (GCSs), where single‐gene knockdown of *UBA5*, *UFC1*, or *UFL1*, as well as UBA5 inhibitor treatment, dramatically impairs the self‐renewal and proliferation capacity of GCSs [167]. PDK1 UFMylation enhances PDK1 ubiquitination and degradation, thereby decreasing phosphorylation and activity of AKT1 and downstream GSK‐3β. Thus, the expression of GSK‐3β‐induced epithelial‐mesenchymal transition (EMT) genes, including vimentin, Snail, and N‐cadherin, is decreased, leading to attenuated metastasis of gastric cancer cells (Figure 5). UFM1 is downregulated in gastric cancer, resulting in elevated expression of these EMT genes and gastric cancer metastasis [168].

### 3.9 UFMylation Participates in Lipid Synthesis and Cytoskeletal Dynamics

Altered lipid metabolism is a hallmark of cancer [169]. Enhanced synthesis or uptake of lipids contributes to cancer cell proliferation and metastasis [170]. Depletion of acyl‐CoA synthetase long chain 3 (ACSL3), which is a rate‐limiting enzyme in lipid synthesis and an important driver of lipid droplet (LD) biogenesis, decreases the stability and anchor of UBA, UFL1, and UFBP1 at the ER membrane in HeLa cells. Additionally, LD formation downregulates the protein levels of UBA5, UFL1, and UFBP1. Therefore, ACSL3 and LD formation are novel regulators of UFMylation [171, 172]. Conversely, UFMylation may modulate lipid synthesis in cancer cells. PI3K‐mTOR‐AKT signaling plays a central role in lipid synthesis by controlling the activity of ATP citrate lyase (ACLY) and the expression of acetyl‐CoA carboxylase 1 (ACC1), fatty acid synthase (FASN), acyl‐CoA synthetase short‐chain family member 2 (ACSS2), stearoyl‐CoA desaturase 1 (SCD1), and 3‐hydroxy‐3‐methylglutaryl‐CoA reductase (HMGCR), which are rate‐limiting enzymes in lipid synthesis [173]. As mentioned above, UFM1 is specifically conjugated to PDK1 but not PI3K or AKT, leading to activated AKT in gastric cancer [168]. In HCC tissues, the expression of both UFL1 and UFBP1 decreased, leading to increased phosphorylation and activity of mTOR and HCC progression (Figure 5) [174]. UFBP1 depletion results in increased lipogenesis and lipid accumulation in hepatocytes by modulating ER stress [175]. Moreover, UFL1 ablation in bovine mammary epithelial cells significantly decreases mTOR phosphorylation and the expression of fat synthesis‐related genes, including FASN, cell death‐inducing DFFA‐like effector A (CIDEA), and ACC1 [176]. Based on these findings, it is rational to predict that UFMylation possibly regulates lipid metabolism in cancer cells. However, the direct connection between UFMylation and lipid metabolism is lacking, and the mechanism of lipid metabolism reprogramming by UFMylation remains uncharted; further investigations are needed to elucidate these important questions.

Recent studies have expanded the repertoire of UFMylation substrates to include cytoskeletal proteins, highlighting their critical roles in mitosis and retinal homeostasis. The UFL1‐UFBP1 complex mediates UFMylation at lysine 1000 of KIF11 (commonly known as Eg5), stabilizing its interaction with microtubules and ensuring proper spindle assembly and accurate chromosome segregation. Loss of KIF11 UFMylation leads to mitotic defects such as multipolar spindles and aneuploidy, which are hallmarks of genomic instability in cancer cells [177]. Another study revealed that UFMylation of KIF11 at lysine 953 regulates its localization to the cilium base, maintaining photoreceptor structure and retinal homeostasis [178, 179, 180]. In cancer cells, KIF11 UFMylation may similarly influence microtubule dynamics during cell migration or invasion, though further validation is required.

## 4 UFMylation in Non‐Neoplastic Diseases

Dysregulation of the UFMylation system has been implicated in a broad spectrum of non‐neoplastic diseases. Loss‐of‐function mutations or deficiencies in UFMylation components are associated with diverse pathological conditions, including neurological disorders, hematopoietic dysfunction, cartilage dysplasia, liver damage, and impairments in other organ systems.

### 4.1 Neurological Disorders

The UFMylation system is essential for the normal development and function of the nervous system in both mice and humans [181]. In mice, cKO of UFM1 in the central nervous system results in neonatal lethality, accompanied by microcephaly and apoptosis of neocortical neurons [55]. Similarly, deletion of UFL1 and UFBP1 in the central nervous system of adult mice leads to microcephaly, neuronal loss, increased neuroinflammation, and, in UFBP1‐deficient mice, epileptiform symptoms [182].

In humans, the UFMylation system is equally critical for nervous system integrity. Mutations in UFM1 are associated with Hypomyelinating Leukodystrophy Type 14, characterized by microcephaly, epilepsy, reduced myelin production, atrophy of the basal ganglia and cerebellum, as well as delayed physical growth and dysplasia. Variants in UBA5 are linked to Developmental and Epileptic Encephalopathy 44 (DEE44), which manifests as global developmental delay, motor impairment, intellectual disability, early‐onset encephalopathy, and recurrent seizures [183, 184]. Additionally, homozygous pathogenic variants in UBA5 can cause fatal congenital neuropathy, while homozygous pathogenic variants in UFC1 result in microcephaly, early infant encephalopathy, and refractory epilepsy, all of which severely compromise individual health [185, 186].

### 4.2 Hematopoietic Dysfunction

The UFMylation system plays a pivotal role in hematopoietic function. In mice, the absence of UBA5 leads to severe anemia, resulting in embryonic lethality [56]. Similarly, loss of UFL1 disrupts the differentiation of megakaryocytes and erythroid progenitors from common myeloid progenitors, impairing hematopoietic development, causing severe anemia, and ultimately leading to death [116]. Furthermore, deficiencies in UFBP1 and CDK5RAP3 result in impaired erythroid function and HSC defects, significantly increasing embryonic mortality [116].

### 4.3 Cartilage Dysplasia

Proper expression of UFBP1 is crucial for the normal development and function of cartilage in both mice and humans. In mice, whole‐body knockout of UFBP1 results in delayed limb bud cartilage condensation [187]. Conditional deletion of UFBP1 in limb mesenchymal cells disrupts growth plate organization, characterized by a shortened proliferative zone and an expanded hypertrophic zone, leading to severe limb shortening and joint abnormalities [17, 188].

In humans, loss‐of‐function mutations in UFBP1 are associated with spondyloepimetaphyseal dysplasia, a condition marked by scoliosis, vertebral compression fractures, flattened vertebrae, and reduced mineralization of long bones, as well as a small trunk, short neck, shortened limbs, and joint laxity [189, 190]. Additionally, UFSP2 is critical for chondrogenesis. Inactivating mutations in UFSP2 lead to Beukes hip dysplasia (BHD), characterized by hip dysplasia [191, 192].

### 4.4 Impairment of the Liver and Other Organs

Multiple components of the UFMylation system are essential for maintaining normal liver function. In mice, the absence of UFL1 and UFBP1 in hepatocytes results in liver damage, increased susceptibility to high‐fat diet (HFD)‐induced fatty liver, spontaneous liver cancer, and diethylnitrosamine (DEN)‐induced hepatocellular carcinoma [174]. Partial loss of CDK5RAP3 in hepatocytes causes severe liver hypoplasia due to delayed proliferation and impaired differentiation [193, 194].

Beyond the liver, the UFMylation system also plays a critical role in other organs. In mice, deletion of UFL1 in cardiomyocytes leads to dilated cardiomyopathy and heart failure [195, 196]. Similarly, loss of UFL1 in the adrenal glands, facial, vestibular cochlear, and cuneiform nerves induces ER stress and activates the PERK signaling pathway in the kidneys, ultimately resulting in renal insufficiency [196].

## 5 Clinical Significance of the UFMylation System in Cancer

The UFMylation system plays critical roles in diverse pathophysiological processes, including aging, development disorders, tumorigenesis, and cancer progression. Given the complex interplay between the UFMylation system and cancer biology, components of this pathway represent promising candidates as both diagnostic biomarkers and therapeutic targets for cancer treatment. Consequently, modulation of the UFMylation system has emerged as an innovative strategy in oncology. Current research has demonstrated the therapeutic potential of targeting this pathway through both in vitro and in vivo studies (Table 2). Developed inhibitors employ distinct mechanistic approaches—either disrupting UFMylation initiation or preventing its termination—with demonstrated efficacy in murine models that underscore the pathway's crucial role in cancer therapeutics.

**TABLE 2:** Targets of the UFMylation system.

Compound	Target	Functions	Stage of development	References
DKM 2‐93	UBA5	Competes with UFM1 to bind the catalytic cysteine 250 (C250) of UBA5, leading to inactive UBA5.	In vivo study	[197]
Adenosine 5'‐sulfamate (ADS)	UBA5	Reacts with the UBA5‐UFM1 thioester to generate a tight‐binding UFM1‐ADS adduct, which associates tightly with and occupies the adenylation site of UBA5 to prevent further substrate binding or catalysis.	In vitro study	[50]
Compound 8.5	UBA5	Inhibits UBA5 through its unique core scaffold incorporating both adenosine and zinc (II)‐cyclen moieties.	In vitro study	[198]
Usenamine A	UBA5	Inhibits UBA5 by potentially binding to its UFM1‐interacting domain, preventing proper complex formation.	In vitro study	[199]
Compound‐8	UFSP2	Inhibits UFSP2 by specifically targeting its active site Cys294, thereby blocking UFM1 deconjugation and enhancing cellular UFMylation.	In vivo study	[135]

### 5.1 The UFMylation System Possesses Potential as a Diagnostic Biomarker or Therapeutic Target

In estrogen receptor‐positive breast cancer, elevated expression of core UFMylation machinery components—UFM1, UBA5, UFC1, UFL1, and UFBP1—correlates strongly with rapid tumor progression and unfavorable outcomes [149], highlighting their potential as dual‐purpose biomarkers for monitoring disease advancement and predicting treatment response. Parallel findings in PDAC demonstrate that UFMylated PIR confers ferroptosis resistance by stabilizing GPX4 [164], with its increased expression associated with aggressive tumor behavior and diminished sensitivity to ferroptosis‐targeted therapies. Notably, in TNBC, UFMylation‐mediated PLAC8 modification drives PD‐L1 overexpression, creating an immunosuppressive tumor microenvironment [133]. This molecular insight suggests that UFMylated PLAC8 detection could enable precise stratification of patients for immune checkpoint blockade. While UFMylation modulators exhibit compelling potential as both diagnostic tools and therapeutic agents, key translational hurdles—particularly regarding isoform specificity and tissue‐selective toxicity—require resolution prior to clinical implementation. Strategic targeting of the UFMylation cascade nevertheless represents a promising avenue for advancing cancer therapeutics

### 5.2 UBA5 Inhibitors

DKM 2‐93 is a covalent inhibitor that selectively modifies the catalytic cysteine (C250) of UBA5, blocking UFM1 activation and subsequent UFMylation. Its specificity was confirmed by isoTOP‐ABPP chemoproteomics, which revealed minimal off‐target effects in pancreatic cancer cells [197]. The anti‐tumor efficacy of DKM 2‐93 was evaluated in nonobese diabetic/severe combined immunodeficiency (NOD‐SCID) mice bearing subcutaneous PaCa2 pancreatic tumor xenografts. Treatment was initiated three days post‐tumor inoculation, with daily intraperitoneal injections administered at a dose of 50 mg/kg. Results demonstrated that DKM 2‐93 significantly suppressed tumor growth compared to vehicle controls, without inducing overt toxicity, as evidenced by the absence of weight loss or organ damage. Target engagement was confirmed via gel‐based activity‐based protein profiling (ABPP), which verified UBA5 inhibition within the tumors and correlated with reduced UFMylation activity. Importantly, the anti‐tumor effects of DKM 2‐93 phenocopied the outcomes observed with shRNA‐mediated UBA5 depletion, further validating UBA5 as a promising therapeutic target in pancreatic cancer. These findings collectively highlight the potential of DKM 2‐93 as a targeted inhibitor for UBA5‐driven tumors.

Additionally, other UBA5 inhibitors have demonstrated regulatory effects on tumor progression; however, research on these compounds remains limited to in vitro studies. Adenosine 5'‐sulfamate (ADS), a pan‐E1 inhibitor, reacts with the UBA5‐UFM1 thioester to generate a tight‐binding UFM1‐ADS adduct that occupies UBA5's adenylation site, effectively suppressing protein UFMylation in HCT116 cells [24, 50]. Compound 8.5 shows remarkable selectivity for UBA5 over other E1 enzymes and 97 human kinases, specifically suppressing proliferation in UBA5‐high cancers like Sk‐Luci6 lung cancer cells [198]. Usenamine A, a lichen‐derived natural product, exhibits anti‐proliferative effects in UBA5‐high breast cancer cells, though its precise mechanism requires further elucidation [199].

### 5.3 UFSP2 Inhibitors

Compound‐8 covalently inhibits UFSP2, the primary deUFMylation enzyme, thereby stabilizing UFMylated substrates like PD‐L1 [135]. The therapeutic potential of UFSP2 inhibition was evaluated in immunocompetent C57BL/6J mice bearing syngeneic MC38 colon tumors. Mice were treated with Compound‐8 either as a single agent or in combination with anti‐PD‐1 antibodies. As a monotherapy, Compound‐8 significantly reduced tumor growth, an effect associated with decreased PD‐L1 levels, further supporting UFSP2's role in PD‐L1 destabilization. Notably, combination therapy with anti‐PD‐1 enhanced tumor suppression beyond either treatment alone, without causing adverse effects such as body weight loss. However, UFSP2's dual role in UFM1 maturation and deconjugation complicates its therapeutic targeting [135].

Notably, inhibitors for other components such as UFL1 and UFC1 remain undeveloped due to technical challenges. Emerging technologies like PROTAC and molecular glues [200, 201] may overcome these limitations by enabling targeted degradation of UFMylation machinery. Given the disease‐modulating roles of UFMylation in various pathological contexts, developing UFMylation agonists represents an equally important therapeutic approach. The dual nature of UFMylation in diseases underscores the need for context‐specific therapeutic strategies targeting this pathway.

## 6 Concluding Remarks

Research over the past several years has elucidated the molecular underpinnings of the UFMylation system and advanced our understanding of its physiological and pathological functions. Despite recent progress, our knowledge about this post‐translational modification remains insufficient. First and foremost, while approximately 30 UFMylation substrates have been discovered, the comprehensive identification of UFMylation substrates is urgently needed to understand the fundamental roles of UFMylation in diseases. To study UFMylation substrates effectively, innovative research methods beyond classical immunoprecipitation and mass spectrometry are essential, necessitating the development of advanced proteomics and biochemical techniques integrated with bioinformatics. A major challenge in the future is to decipher which and how upstream signals or regulators are engaged in the UFMylation system, and the contexts of their action. So far, only ATM and AMPK have been reported to phosphorylate UFL1 in response to DNA damage and glucose deprivation, respectively [118, 136]. However, it remains unclear how the UFMylation system senses and responds to the exogenous and endogenous stimuli. High‐throughput screening using CRISPR or shRNA may uncover potential upstream regulators.

Another challenge is to elucidate the complicated crosstalk between UFMylation and other PTMs. While ubiquitination and phosphorylation dominate the PTM landscape, UFMylation represents a specialized Ub‐like modification with unique mechanistic and functional features. Unlike ubiquitination, which forms diverse polyubiquitin chains to regulate protein degradation, signaling, or localization [2], UFMylation primarily generates mono‐UFM1 conjugates or short K69‐linked chains [44]. This structural simplicity suggests distinct regulatory roles, potentially fine‐tuning substrate stability or interactions rather than targeting proteins for proteasomal degradation. Moreover, UFMylation exhibits compartmentalized activity, predominantly localizing to the ER and nucleus [24, 67], whereas ubiquitination operates ubiquitously across cellular compartments. This spatial restriction may explain UFMylation's critical roles in ER‐phagy, ribosomal quality control, and DNA damage repair processes where ubiquitination plays complementary but non‐redundant roles [17, 20, 118]. For instance, while ubiquitination marks misfolded ER proteins for proteasomal degradation via ERAD [86], UFMylation resolves ribosomal stalling and ER stress through lysosomal clearance [79, 89], highlighting a division of labor within PTM networks. Notably, UFMylation crosstalks with other PTMs. It antagonizes ubiquitination by shielding substrates (e.g., ERα and p53) from E3 ligases [128, 202], yet enhances ubiquitination of PD‐L1 and MAVS to promote their degradation [135, 137]. Such context‐dependent interplay underscores UFMylation's role as a molecular “switch” that integrates cellular signals beyond canonical Ub pathways. Future studies should explore how UFMylation coordinates with SUMOylation or acetylation, as suggested by UBA5's nuclear translocation with SUMO2 [59]. UFMylation also influences protein phosphorylation, such as PERK, ATM, and mTOR. It remains unexplored whether these kinases phosphorylate components of the UFMylation system to form a regulatory loop. The effect of UFMylation on other PTMs remains to be investigated.

One important question to be addressed is how UFMylation code is “read” and interpreted by cells. UFL1, the unique E3 ligase in the UFMylation system, corresponds to the “writer” of the code that generates mono‐or poly‐UFMylation on various substrates. UFSP1 and UFSP2 “erase” the code. However, it remains unclear why the UFMylation system has different or even opposite effects on specific cellular or molecular processes in different cell types. One possible explanation is that there must exist multiple UFMylation “readers” to direct particular physiological or pathological outcomes. Recent studies have demonstrated that the UFL1‐UFBP1‐UFM1‐CDK5RAP3 complex and SAYSD1 function as potential UFMylation “readers” [81, 82, 83]. Hence, identification of novel UFMylation “readers” is an important direction in UFMylation research in the future. Finally, attention should be paid to the UFMylation‐independent functions of the UFMylation system. For example, UFL1 maintains STING expression and protein sorting at the ER independent of UFMylation [84, 139].

## Author Contributions

**Yijie Wang**: conceptualization, funding acquisition, methodology, supervision, writing – original draft, writing – review and editing. **Changliang Shan**: conceptualization, funding acquisition, methodology, supervision, writing – original draft, writing – review and editing. **Yan Chen**: conceptualization, funding acquisition, methodology, supervision, writing – original draft, writing – review and editing. **Huiyan Li**: writing – original draft, writing – review and editing. **Fei Meng**: writing – original draft, writing – review and editing. **Junjie Liang**: writing – original draft, writing – review and editing. All authors have read and approved the final manuscript.

## Ethics Statement

The authors have nothing to report.

## Conflicts of Interest

The authors declare no conflicts of interest.

## Data Availability

The authors have nothing to report.
